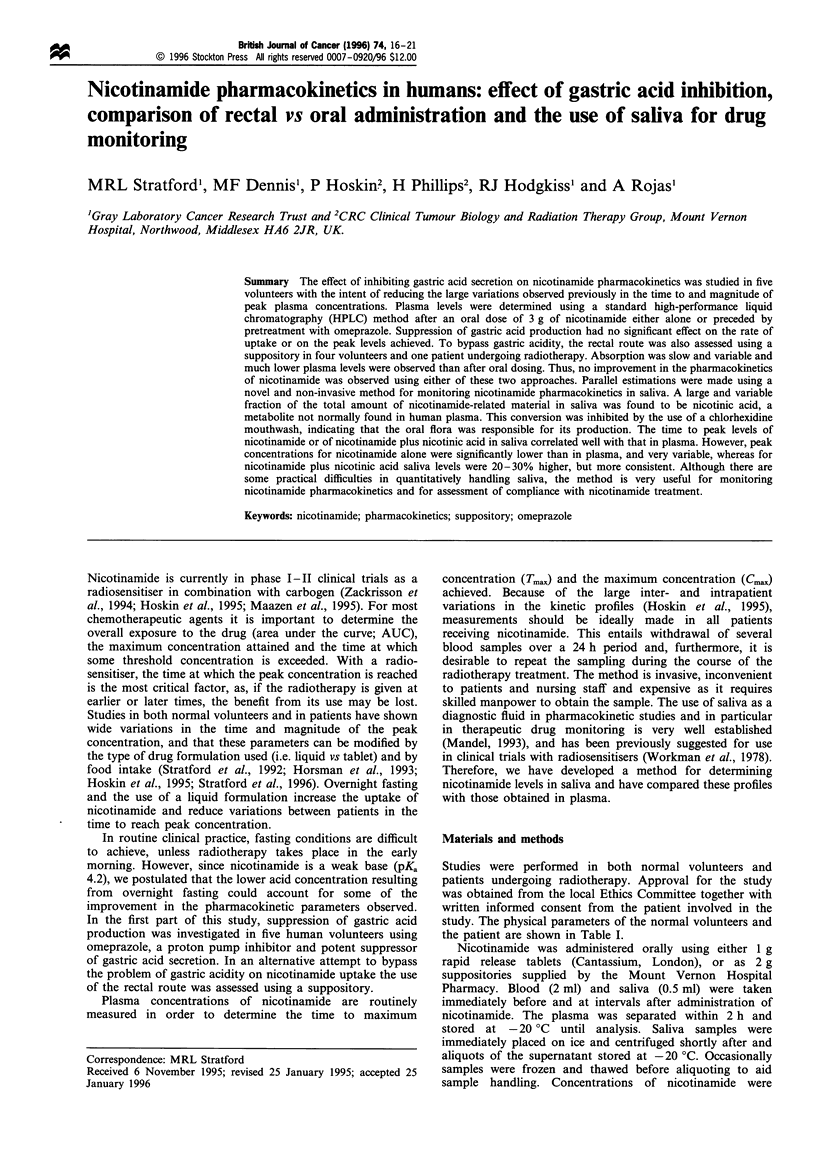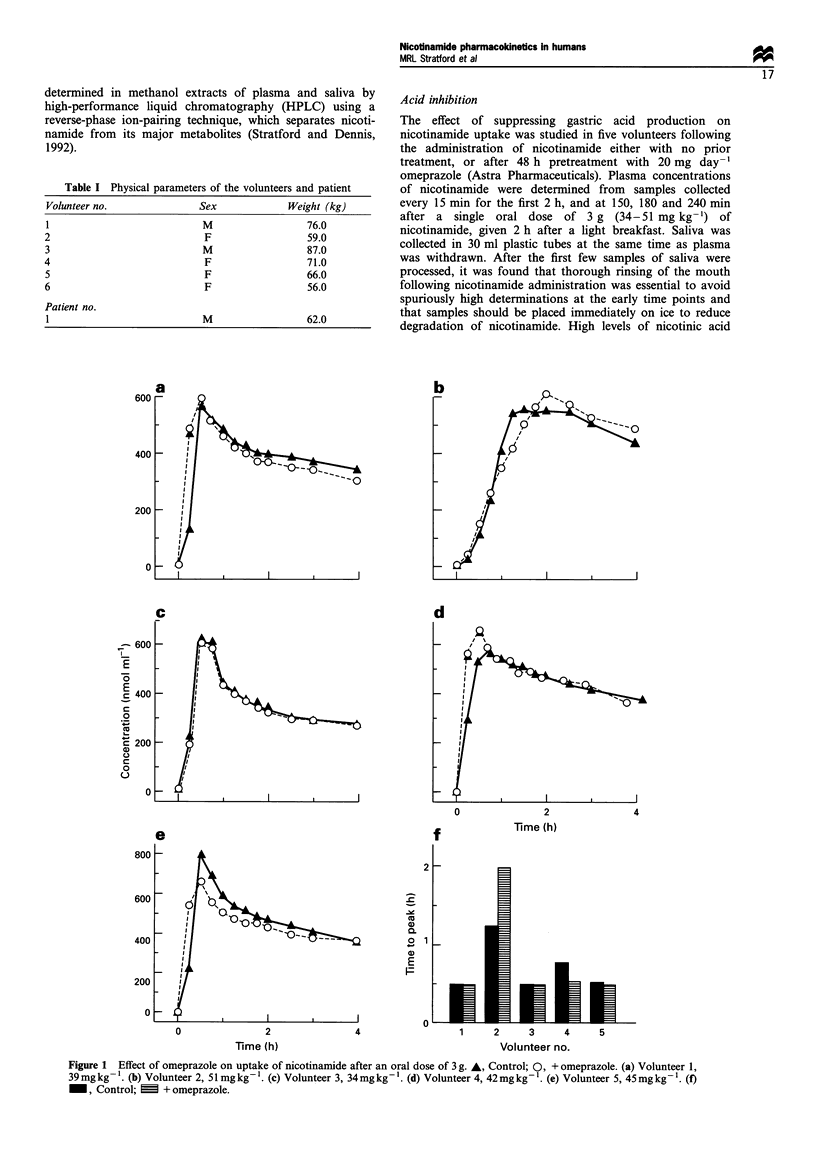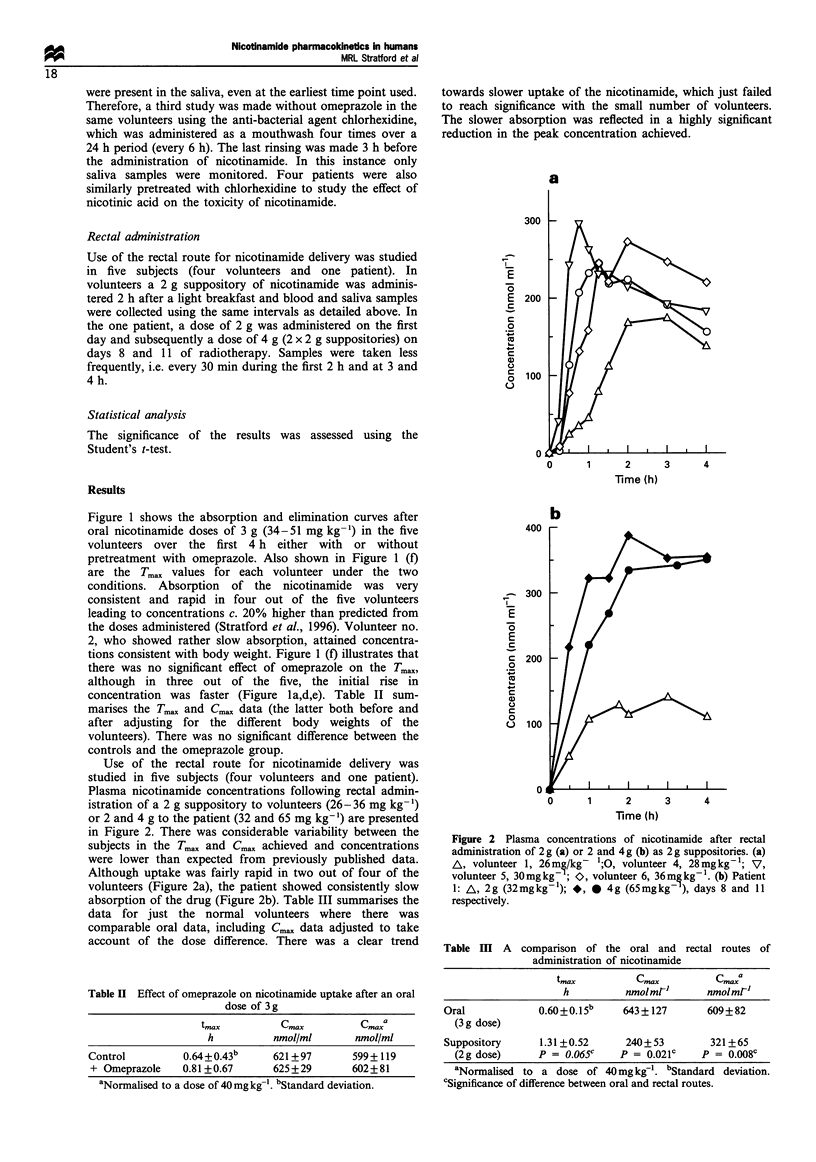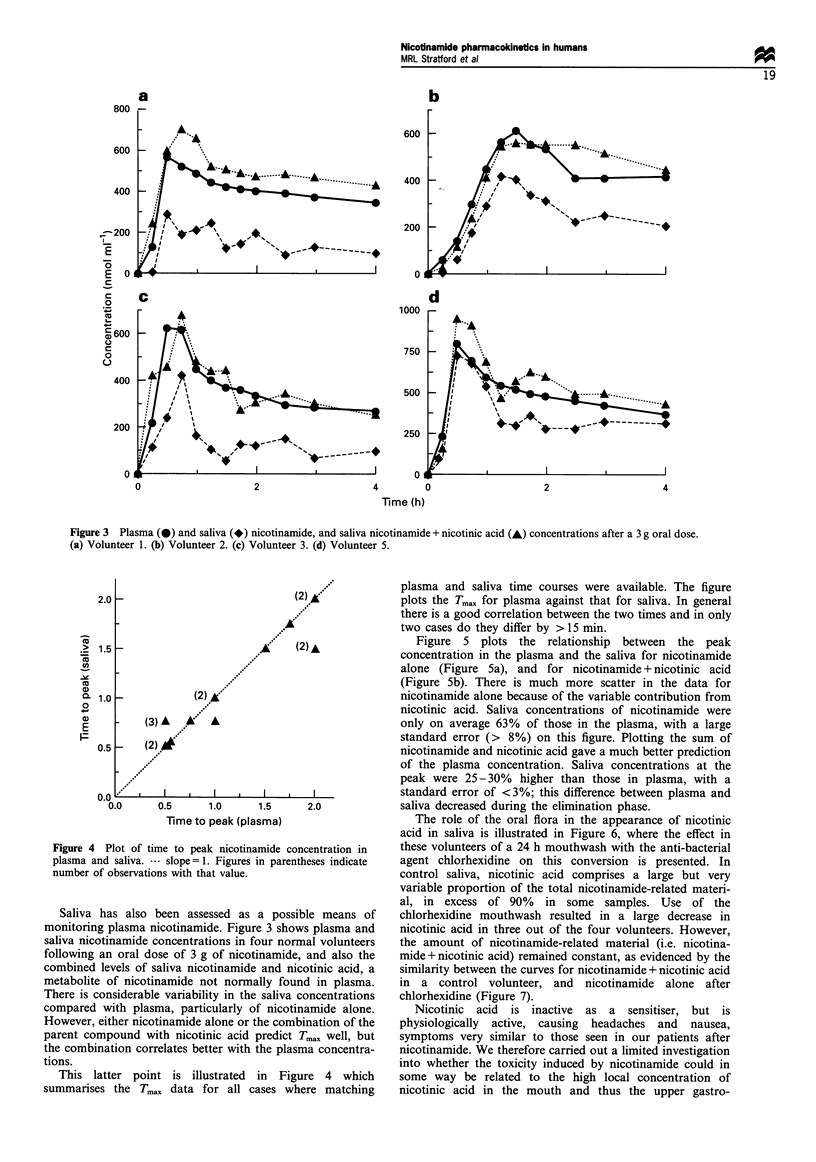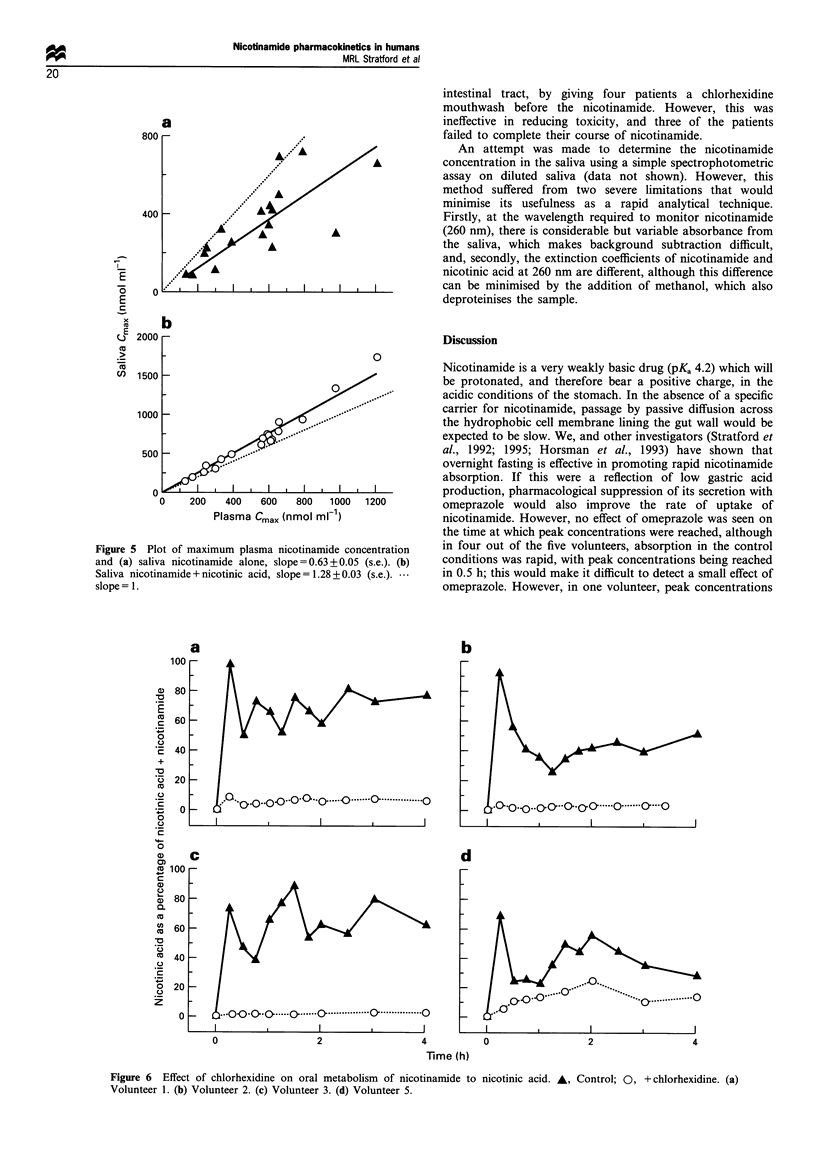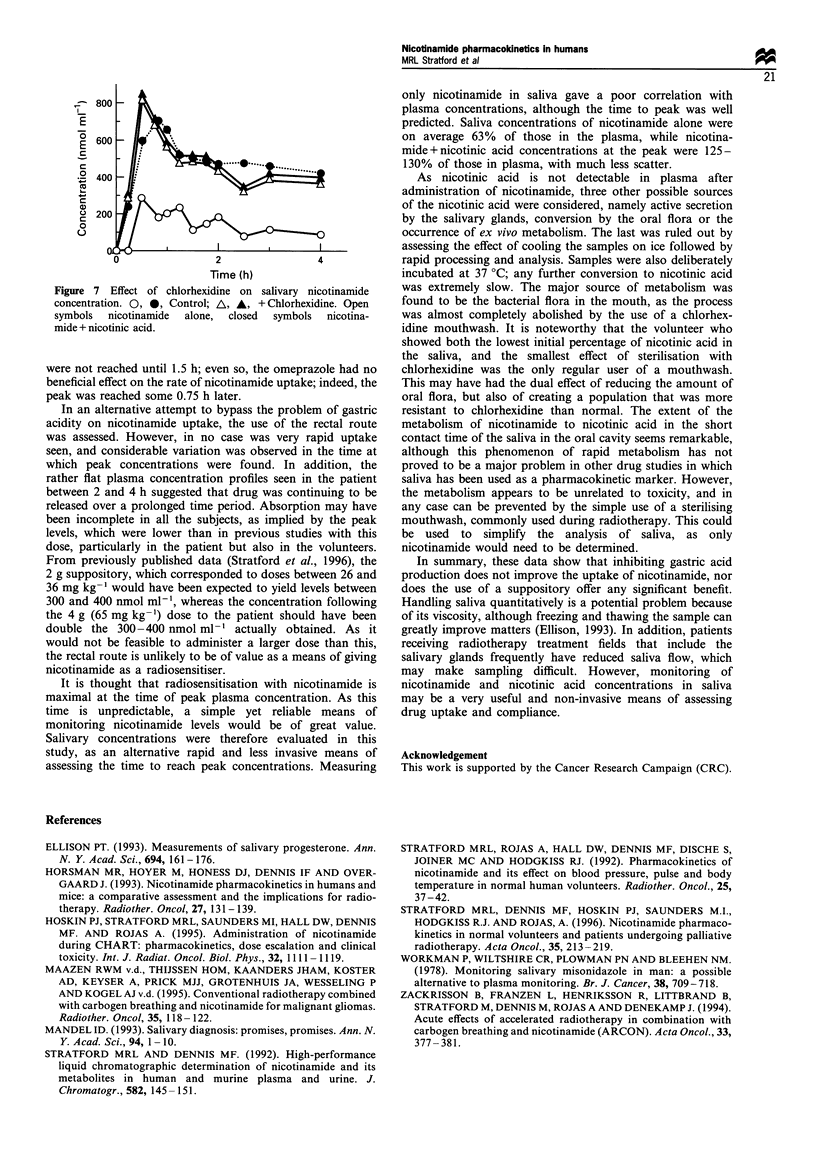# Nicotinamide pharmacokinetics in humans: effect of gastric acid inhibition, comparison of rectal vs oral administration and the use of saliva for drug monitoring.

**DOI:** 10.1038/bjc.1996.309

**Published:** 1996-07

**Authors:** M. R. Stratford, M. F. Dennis, P. Hoskin, H. Phillips, R. J. Hodgkiss, A. Rojas

**Affiliations:** Gray Laboratory Cancer Research Trust, Mount Vernon Hospital, Northwood, Middlesex, UK.

## Abstract

The effect of inhibiting gastric acid secretion on nicotinamide pharmacokinetics was studied in five volunteers with the intent of reducing the large variations observed previously in the time to and magnitude of peak plasma concentrations. Plasma levels were determined using a standard high-performance liquid chromatography (HPLC) method after an oral dose of 3 g of nicotinamide either alone or preceded by pretreatment with omeprazole. Suppression of gastric acid production had no significant effect on the rate of uptake or on the peak levels achieved. To bypass gastric acidity, the rectal route was also assessed using a suppository in four volunteers and one patient undergoing radiotherapy. Absorption was slow and variable and much lower plasma levels were observed than after oral dosing. Thus, no improvement in the pharmacokinetics of nicotinamide was observed using either of these two approaches. Parallel estimations were made using a novel and non-invasive method for monitoring nicotinamide pharmacokinetics in saliva. A large and variable fraction of the total amount of nicotinamide-related material in saliva was found to be nicotinic acid, a metabolite not normally found in human plasma. This conversion was inhibited by the use of a chlorhexidine mouthwash, indicating that the oral flora was responsible for its production. The time to peak levels of nicotinamide or of nicotinamide plus nicotinic acid in saliva correlated well with that in plasma. However, peak concentrations for nicotinamide alone were significantly lower than in plasma, and very variable, whereas for nicotinamide plus nicotinic acid saliva levels were 20-30% higher, but more consistent. Although there are some practical difficulties in quantitatively handling saliva, the method is very useful for monitoring nicotinamide pharmacokinetics and for assessment of compliance with nicotinamide treatment.


British Journal of Cancer (1996) 74, 16-21
© 1996 Stockton Press All rights reserved 0007-0920/96 $12.00

### Nicotinamide pharmacokinetics in humans: effect of gastric acid inhibition, comparison of rectal *vs* oral administration and the use of saliva for drug monitoring

MRL Stratford[1], MF Dennis[1], P Hoskin[2], H Phillips[2], RJ Hodgkiss[1] and A Rojas[1]

[1]*Gray Laboratory Cancer Research Trust and* [2]*CRC Clinical Tumour Biology and Radiation Therapy Group, Mount Vernon Hospital, Northwood, Middlesex HA6 2JR, UK.*

**Summary** The effect of inhibiting gastric acid secretion on nicotinamide pharmacokinetics was studied in five volunteers with the intent of reducing the large variations observed previously in the time to and magnitude of peak plasma concentrations. Plasma levels were determined using a standard high-performance liquid chromatography (HPLC) method after an oral dose of 3 g of nicotinamide either alone or preceded by pretreatment with omeprazole. Suppression of gastric acid production had no significant effect on the rate of uptake or on the peak levels achieved. To bypass gastric acidity, the rectal route was also assessed using a suppository in four volunteers and one patient undergoing radiotherapy. Absorption was slow and variable and much lower plasma levels were observed than after oral dosing. Thus, no improvement in the pharmacokinetics of nicotinamide was observed using either of these two approaches. Parallel estimations were made using a novel and non-invasive method for monitoring nicotinamide pharmacokinetics in saliva. A large and variable fraction of the total amount of nicotinamide-related material in saliva was found to be nicotinic acid, a metabolite not normally found in human plasma. This conversion was inhibited by the use of a chlorhexidine mouthwash, indicating that the oral flora was responsible for its production. The time to peak levels of nicotinamide or of nicotinamide plus nicotinic acid in saliva correlated well with that in plasma. However, peak concentrations for nicotinamide alone were significantly lower than in plasma, and very variable, whereas for nicotinamide plus nicotinic acid saliva levels were 20–30% higher, but more consistent. Although there are some practical difficulties in quantitatively handling saliva, the method is very useful for monitoring nicotinamide pharmacokinetics and for assessment of compliance with nicotinamide treatment.

**Keywords:** nicotinamide; pharmacokinetics; suppository; omeprazole

Nicotinamide is currently in phase I–II clinical trials as a radiosensitiser in combination with carbogen (Zackrisson *et al.*, 1994; Hoskin *et al.*, 1995; Maazen *et al.*, 1995). For most chemotherapeutic agents it is important to determine the overall exposure to the drug (area under the curve; AUC), the maximum concentration attained and the time at which some threshold concentration is exceeded. With a radiosensitiser, the time at which the peak concentration is reached is the most critical factor, as, if the radiotherapy is given at earlier or later times, the benefit from its use may be lost. Studies in both normal volunteers and in patients have shown wide variations in the time and magnitude of the peak concentration, and that these parameters can be modified by the type of drug formulation used (i.e. liquid *vs* tablet) and by food intake (Stratford *et al.*, 1992; Horsman *et al.*, 1993; Hoskin *et al.*, 1995; Stratford *et al.*, 1996). Overnight fasting and the use of a liquid formulation increase the uptake of nicotinamide and reduce variations between patients in the time to reach peak concentration.

In routine clinical practice, fasting conditions are difficult to achieve, unless radiotherapy takes place in the early morning. However, since nicotinamide is a weak base (p$K_a$ 4.2), we postulated that the lower acid concentration resulting from overnight fasting could account for some of the improvement in the pharmacokinetic parameters observed. In the first part of this study, suppression of gastric acid production was investigated in five human volunteers using omeprazole, a proton pump inhibitor and potent suppressor of gastric acid secretion. In an alternative attempt to bypass the problem of gastric acidity on nicotinamide uptake the use of the rectal route was assessed using a suppository.

Plasma concentrations of nicotinamide are routinely measured in order to determine the time to maximum concentration ($T_{max}$) and the maximum concentration ($C_{max}$) achieved. Because of the large inter- and intrapatient variations in the kinetic profiles (Hoskin *et al.*, 1995), measurements should be ideally made in all patients receiving nicotinamide. This entails withdrawal of several blood samples over a 24 h period and, furthermore, it is desirable to repeat the sampling during the course of the radiotherapy treatment. The method is invasive, inconvenient to patients and nursing staff and expensive as it requires skilled manpower to obtain the sample. The use of saliva as a diagnostic fluid in pharmacokinetic studies and in particular in therapeutic drug monitoring is very well established (Mandel, 1993), and has been previously suggested for use in clinical trials with radiosensitisers (Workman *et al.*, 1978). Therefore, we have developed a method for determining nicotinamide levels in saliva and have compared these profiles with those obtained in plasma.

### Materials and methods

Studies were performed in both normal volunteers and patients undergoing radiotherapy. Approval for the study was obtained from the local Ethics Committee together with written informed consent from the patient involved in the study. The physical parameters of the normal volunteers and the patient are shown in Table I.

Nicotinamide was administered orally using either 1 g rapid release tablets (Cantassium, London), or as 2 g suppositories supplied by the Mount Vernon Hospital Pharmacy. Blood (2 ml) and saliva (0.5 ml) were taken immediately before and at intervals after administration of nicotinamide. The plasma was separated within 2 h and stored at −20 °C until analysis. Saliva samples were immediately placed on ice and centrifuged shortly after and aliquots of the supernatant stored at −20 °C. Occasionally samples were frozen and thawed before aliquoting to aid sample handling. Concentrations of nicotinamide were

Correspondence: MRL Stratford
Received 6 November 1995; revised 25 January 1995; accepted 25 January 1996

determined in methanol extracts of plasma and saliva by high-performance liquid chromatography (HPLC) using a reverse-phase ion-pairing technique, which separates nicotinamide from its major metabolites (Stratford and Dennis, 1992).

**Table I** Physical parameters of the volunteers and patient

| *Volunteer no.* | *Sex* | *Weight (kg)* |
|---|---|---|
| 1 | M | 76.0 |
| 2 | F | 59.0 |
| 3 | M | 87.0 |
| 4 | F | 71.0 |
| 5 | F | 66.0 |
| 6 | F | 56.0 |
| *Patient no.* | | |
| 1 | M | 62.0 |

*Acid inhibition*

The effect of suppressing gastric acid production on nicotinamide uptake was studied in five volunteers following the administration of nicotinamide either with no prior treatment, or after 48 h pretreatment with 20 mg $day^{-1}$ omeprazole (Astra Pharmaceuticals). Plasma concentrations of nicotinamide were determined from samples collected every 15 min for the first 2 h, and at 150, 180 and 240 min after a single oral dose of 3 g (34–51 mg $kg^{-1}$) of nicotinamide, given 2 h after a light breakfast. Saliva was collected in 30 ml plastic tubes at the same time as plasma was withdrawn. After the first few samples of saliva were processed, it was found that thorough rinsing of the mouth following nicotinamide administration was essential to avoid spuriously high determinations at the early time points and that samples should be placed immediately on ice to reduce degradation of nicotinamide. High levels of nicotinic acid

**Figure 1** Effect of omeprazole on uptake of nicotinamide after an oral dose of 3 g. ▲, Control; ○, + omeprazole. (**a**) Volunteer 1, 39 mg $kg^{-1}$. (**b**) Volunteer 2, 51 mg $kg^{-1}$. (**c**) Volunteer 3, 34 mg $kg^{-1}$. (**d**) Volunteer 4, 42 mg $kg^{-1}$. (**e**) Volunteer 5, 45 mg $kg^{-1}$. (**f**) ■, Control; ▤ + omeprazole.

were present in the saliva, even at the earliest time point used. Therefore, a third study was made without omeprazole in the same volunteers using the anti-bacterial agent chlorhexidine, which was administered as a mouthwash four times over a 24 h period (every 6 h). The last rinsing was made 3 h before the administration of nicotinamide. In this instance only saliva samples were monitored. Four patients were also similarly pretreated with chlorhexidine to study the effect of nicotinic acid on the toxicity of nicotinamide.

*Rectal administration*

Use of the rectal route for nicotinamide delivery was studied in five subjects (four volunteers and one patient). In volunteers a 2 g suppository of nicotinamide was administered 2 h after a light breakfast and blood and saliva samples were collected using the same intervals as detailed above. In the one patient, a dose of 2 g was administered on the first day and subsequently a dose of 4 g (2 x 2 g suppositories) on days 8 and 11 of radiotherapy. Samples were taken less frequently, i.e. every 30 min during the first 2 h and at 3 and 4 h.

*Statistical analysis*

The significance of the results was assessed using the Student's *t*-test.

### Results

Figure 1 shows the absorption and elimination curves after oral nicotinamide doses of 3 g (34–51 mg $kg^{-1}$) in the five volunteers over the first 4 h either with or without pretreatment with omeprazole. Also shown in Figure 1 (f) are the $T_{max}$ values for each volunteer under the two conditions. Absorption of the nicotinamide was very consistent and rapid in four out of the five volunteers leading to concentrations *c*. 20% higher than predicted from the doses administered (Stratford *et al*., 1996). Volunteer no. 2, who showed rather slow absorption, attained concentrations consistent with body weight. Figure 1 (f) illustrates that there was no significant effect of omeprazole on the $T_{max}$, although in three out of the five, the initial rise in concentration was faster (Figure 1a,d,e). Table II summarises the $T_{max}$ and $C_{max}$ data (the latter both before and after adjusting for the different body weights of the volunteers). There was no significant difference between the controls and the omeprazole group.

Use of the rectal route for nicotinamide delivery was studied in five subjects (four volunteers and one patient). Plasma nicotinamide concentrations following rectal administration of a 2 g suppository to volunteers (26–36 mg $kg^{-1}$) or 2 and 4 g to the patient (32 and 65 mg $kg^{-1}$) are presented in Figure 2. There was considerable variability between the subjects in the $T_{max}$ and $C_{max}$ achieved and concentrations were lower than expected from previously published data. Although uptake was fairly rapid in two out of four of the volunteers (Figure 2a), the patient showed consistently slow absorption of the drug (Figure 2b). Table III summarises the data for just the normal volunteers where there was comparable oral data, including $C_{max}$ data adjusted to take account of the dose difference. There was a clear trend towards slower uptake of the nicotinamide, which just failed to reach significance with the small number of volunteers. The slower absorption was reflected in a highly significant reduction in the peak concentration achieved.

**Table II** Effect of omeprazole on nicotinamide uptake after an oral dose of 3 g

| | $t_{max}$ h | $C_{max}$ nmol/ml | $C_{max}$[a] nmol/ml |
|---|---|---|---|
| Control | 0.64±0.43[b] | 621±97 | 599±119 |
| + Omeprazole | 0.81±0.67 | 625±29 | 602±81 |

[a]Normalised to a dose of 40 mg $kg^{-1}$. [b]Standard deviation.

**Figure 2** Plasma concentrations of nicotinamide after rectal administration of 2 g (**a**) or 2 and 4 g (**b**) as 2 g suppositories. (**a**) △, volunteer 1, 26 mg/$kg^{-1}$; O, volunteer 4, 28 mg $kg^{-1}$; ▽, volunteer 5, 30 mg $kg^{-1}$; ◇, volunteer 6, 36 mg $kg^{-1}$. (**b**) Patient 1: △, 2 g (32 mg $kg^{-1}$); ◆, ● 4 g (65 mg $kg^{-1}$), days 8 and 11 respectively.

**Table III** A comparison of the oral and rectal routes of administration of nicotinamide

| | $t_{max}$ h | $C_{max}$ nmol $ml^{-1}$ | $C_{max}$[a] nmol $ml^{-1}$ |
|---|---|---|---|
| Oral (3 g dose) | 0.60±0.15[b] | 643±127 | 609±82 |
| Suppository (2 g dose) | 1.31±0.52 $P$ = 0.065[c] | 240±53 $P$ = 0.021[c] | 321±65 $P$ = 0.008[c] |

[a]Normalised to a dose of 40 mg $kg^{-1}$. [b]Standard deviation. [c]Significance of difference between oral and rectal routes.

**Figure 3** Plasma (●) and saliva (◆) nicotinamide, and saliva nicotinamide + nicotinic acid (▲) concentrations after a 3 g oral dose. (**a**) Volunteer 1. (**b**) Volunteer 2. (**c**) Volunteer 3. (**d**) Volunteer 5.

**Figure 4** Plot of time to peak nicotinamide concentration in plasma and saliva. ··· slope = 1. Figures in parentheses indicate number of observations with that value.

Saliva has also been assessed as a possible means of monitoring plasma nicotinamide. Figure 3 shows plasma and saliva nicotinamide concentrations in four normal volunteers following an oral dose of 3 g of nicotinamide, and also the combined levels of saliva nicotinamide and nicotinic acid, a metabolite of nicotinamide not normally found in plasma. There is considerable variability in the saliva concentrations compared with plasma, particularly of nicotinamide alone. However, either nicotinamide alone or the combination of the parent compound with nicotinic acid predict $T_{max}$ well, but the combination correlates better with the plasma concentrations.

This latter point is illustrated in Figure 4 which summarises the $T_{max}$ data for all cases where matching plasma and saliva time courses were available. The figure plots the $T_{max}$ for plasma against that for saliva. In general there is a good correlation between the two times and in only two cases do they differ by >15 min.

Figure 5 plots the relationship between the peak concentration in the plasma and the saliva for nicotinamide alone (Figure 5a), and for nicotinamide + nicotinic acid (Figure 5b). There is much more scatter in the data for nicotinamide alone because of the variable contribution from nicotinic acid. Saliva concentrations of nicotinamide were only on average 63% of those in the plasma, with a large standard error (> 8%) on this figure. Plotting the sum of nicotinamide and nicotinic acid gave a much better prediction of the plasma concentration. Saliva concentrations at the peak were 25–30% higher than those in plasma, with a standard error of <3%; this difference between plasma and saliva decreased during the elimination phase.

The role of the oral flora in the appearance of nicotinic acid in saliva is illustrated in Figure 6, where the effect in these volunteers of a 24 h mouthwash with the anti-bacterial agent chlorhexidine on this conversion is presented. In control saliva, nicotinic acid comprises a large but very variable proportion of the total nicotinamide-related material, in excess of 90% in some samples. Use of the chlorhexidine mouthwash resulted in a large decrease in nicotinic acid in three out of the four volunteers. However, the amount of nicotinamide-related material (i.e. nicotinamide + nicotinic acid) remained constant, as evidenced by the similarity between the curves for nicotinamide + nicotinic acid in a control volunteer, and nicotinamide alone after chlorhexidine (Figure 7).

Nicotinic acid is inactive as a sensitiser, but is physiologically active, causing headaches and nausea, symptoms very similar to those seen in our patients after nicotinamide. We therefore carried out a limited investigation into whether the toxicity induced by nicotinamide could in some way be related to the high local concentration of nicotinic acid in the mouth and thus the upper gastro-

**Figure 5** Plot of maximum plasma nicotinamide concentration and (**a**) saliva nicotinamide alone, slope = 0.63 ± 0.05 (s.e.). (**b**) Saliva nicotinamide + nicotinic acid, slope = 1.28 ± 0.03 (s.e.). ··· slope = 1.

intestinal tract, by giving four patients a chlorhexidine mouthwash before the nicotinamide. However, this was ineffective in reducing toxicity, and three of the patients failed to complete their course of nicotinamide.

An attempt was made to determine the nicotinamide concentration in the saliva using a simple spectrophotometric assay on diluted saliva (data not shown). However, this method suffered from two severe limitations that would minimise its usefulness as a rapid analytical technique. Firstly, at the wavelength required to monitor nicotinamide (260 nm), there is considerable but variable absorbance from the saliva, which makes background subtraction difficult, and, secondly, the extinction coefficients of nicotinamide and nicotinic acid at 260 nm are different, although this difference can be minimised by the addition of methanol, which also deproteinises the sample.

### Discussion

Nicotinamide is a very weakly basic drug (p$K_a$ 4.2) which will be protonated, and therefore bear a positive charge, in the acidic conditions of the stomach. In the absence of a specific carrier for nicotinamide, passage by passive diffusion across the hydrophobic cell membrane lining the gut wall would be expected to be slow. We, and other investigators (Stratford *et al.*, 1992; 1995; Horsman *et al.*, 1993) have shown that overnight fasting is effective in promoting rapid nicotinamide absorption. If this were a reflection of low gastric acid production, pharmacological suppression of its secretion with omeprazole would also improve the rate of uptake of nicotinamide. However, no effect of omeprazole was seen on the time at which peak concentrations were reached, although in four out of the five volunteers, absorption in the control conditions was rapid, with peak concentrations being reached in 0.5 h; this would make it difficult to detect a small effect of omeprazole. However, in one volunteer, peak concentrations

**Figure 6** Effect of chlorhexidine on oral metabolism of nicotinamide to nicotinic acid. ▲, Control; ○, + chlorhexidine. (**a**) Volunteer 1. (**b**) Volunteer 2. (**c**) Volunteer 3. (**d**) Volunteer 5.

**Figure 7** Effect of chlorhexidine on salivary nicotinamide concentration. ○, ●, Control; △, ▲, + Chlorhexidine. Open symbols nicotinamide alone, closed symbols nicotinamide + nicotinic acid.

were not reached until 1.5 h; even so, the omeprazole had no beneficial effect on the rate of nicotinamide uptake; indeed, the peak was reached some 0.75 h later.

In an alternative attempt to bypass the problem of gastric acidity on nicotinamide uptake, the use of the rectal route was assessed. However, in no case was very rapid uptake seen, and considerable variation was observed in the time at which peak concentrations were found. In addition, the rather flat plasma concentration profiles seen in the patient between 2 and 4 h suggested that drug was continuing to be released over a prolonged time period. Absorption may have been incomplete in all the subjects, as implied by the peak levels, which were lower than in previous studies with this dose, particularly in the patient but also in the volunteers. From previously published data (Stratford *et al.*, 1996), the 2 g suppository, which corresponded to doses between 26 and 36 mg kg$^{-1}$ would have been expected to yield levels between 300 and 400 nmol ml$^{-1}$, whereas the concentration following the 4 g (65 mg kg$^{-1}$) dose to the patient should have been double the 300–400 nmol ml$^{-1}$ actually obtained. As it would not be feasible to administer a larger dose than this, the rectal route is unlikely to be of value as a means of giving nicotinamide as a radiosensitiser.

It is thought that radiosensitisation with nicotinamide is maximal at the time of peak plasma concentration. As this time is unpredictable, a simple yet reliable means of monitoring nicotinamide levels would be of great value. Salivary concentrations were therefore evaluated in this study, as an alternative rapid and less invasive means of assessing the time to reach peak concentrations. Measuring only nicotinamide in saliva gave a poor correlation with plasma concentrations, although the time to peak was well predicted. Saliva concentrations of nicotinamide alone were on average 63% of those in the plasma, while nicotinamide + nicotinic acid concentrations at the peak were 125–130% of those in plasma, with much less scatter.

As nicotinic acid is not detectable in plasma after administration of nicotinamide, three other possible sources of the nicotinic acid were considered, namely active secretion by the salivary glands, conversion by the oral flora or the occurrence of *ex vivo* metabolism. The last was ruled out by assessing the effect of cooling the samples on ice followed by rapid processing and analysis. Samples were also deliberately incubated at 37 °C; any further conversion to nicotinic acid was extremely slow. The major source of metabolism was found to be the bacterial flora in the mouth, as the process was almost completely abolished by the use of a chlorhexidine mouthwash. It is noteworthy that the volunteer who showed both the lowest initial percentage of nicotinic acid in the saliva, and the smallest effect of sterilisation with chlorhexidine was the only regular user of a mouthwash. This may have had the dual effect of reducing the amount of oral flora, but also of creating a population that was more resistant to chlorhexidine than normal. The extent of the metabolism of nicotinamide to nicotinic acid in the short contact time of the saliva in the oral cavity seems remarkable, although this phenomenon of rapid metabolism has not proved to be a major problem in other drug studies in which saliva has been used as a pharmacokinetic marker. However, the metabolism appears to be unrelated to toxicity, and in any case can be prevented by the simple use of a sterilising mouthwash, commonly used during radiotherapy. This could be used to simplify the analysis of saliva, as only nicotinamide would need to be determined.

In summary, these data show that inhibiting gastric acid production does not improve the uptake of nicotinamide, nor does the use of a suppository offer any significant benefit. Handling saliva quantitatively is a potential problem because of its viscosity, although freezing and thawing the sample can greatly improve matters (Ellison, 1993). In addition, patients receiving radiotherapy treatment fields that include the salivary glands frequently have reduced saliva flow, which may make sampling difficult. However, monitoring of nicotinamide and nicotinic acid concentrations in saliva may be a very useful and non-invasive means of assessing drug uptake and compliance.

**Acknowledgement**
This work is supported by the Cancer Research Campaign (CRC).

### References

ELLISON PT. (1993). Measurements of salivary progesterone. *Ann. N. Y. Acad. Sci.*, **694**, 161–176.

HORSMAN MR, HOYER M, HONESS DJ, DENNIS IF AND OVERGAARD J. (1993). Nicotinamide pharmacokinetics in humans and mice: a comparative assessment and the implications for radiotherapy. *Radiother. Oncol*, **27**, 131–139.

HOSKIN PJ, STRATFORD MRL, SAUNDERS MI, HALL DW, DENNIS MF. AND ROJAS A. (1995). Administration of nicotinamide during CHART: pharmacokinetics, dose escalation and clinical toxicity. *Int. J. Radiat. Oncol. Biol. Phys.*, **32**, 1111–1119.

MAAZEN RWM v.d., THIJSSEN HOM, KAANDERS JHAM, KOSTER AD, KEYSER A, PRICK MJJ, GROTENHUIS JA, WESSELING P AND KOGEL AJ v.d. (1995). Conventional radiotherapy combined with carbogen breathing and nicotinamide for malignant gliomas. *Radiother. Oncol*, **35**, 118–122.

MANDEL ID. (1993). Salivary diagnosis: promises, promises. *Ann. N. Y. Acad. Sci.*, **94**, 1–10.

STRATFORD MRL AND DENNIS MF. (1992). High-performance liquid chromatographic determination of nicotinamide and its metabolites in human and murine plasma and urine. *J. Chromatogr.*, **582**, 145–151.

STRATFORD MRL, ROJAS A, HALL DW, DENNIS MF, DISCHE S, JOINER MC AND HODGKISS RJ. (1992). Pharmacokinetics of nicotinamide and its effect on blood pressure, pulse and body temperature in normal human volunteers. *Radiother. Oncol.*, **25**, 37–42.

STRATFORD MRL, DENNIS MF, HOSKIN PJ, SAUNDERS M.I., HODGKISS R.J. AND ROJAS, A. (1996). Nicotinamide pharmacokinetics in normal volunteers and patients undergoing palliative radiotherapy. *Acta Oncol.*, **35**, 213–219.

WORKMAN P, WILTSHIRE CR, PLOWMAN PN AND BLEEHEN NM. (1978). Monitoring salivary misonidazole in man: a possible alternative to plasma monitoring. *Br. J. Cancer*, **38**, 709–718.

ZACKRISSON B, FRANZEN L, HENRIKSSON R, LITTBRAND B, STRATFORD M, DENNIS M, ROJAS A AND DENEKAMP J. (1994). Acute effects of accelerated radiotherapy in combination with carbogen breathing and nicotinamide (ARCON). *Acta Oncol.*, **33**, 377-381.